# Cross-Border Higher Education: The Expansion of International Branch Campuses

**DOI:** 10.1007/s11162-022-09674-y

**Published:** 2022-01-17

**Authors:** Jordi Paniagua, Cristina Villó, Maria Escrivà-Beltran

**Affiliations:** 1 University of Valencia, Av. Tarongers s/n, 46022 Valencia, Spain; 2 Kellogg Institute, University of Notre Dame, Notre Dame, USA; 3 Escuela de Doctorado, Catholic University of Valencia, Valencia, Spain

**Keywords:** International branch campuses, Higher education, Foreign direct investment, Gravity equation

## Abstract

The international expansion of higher education has intensified in recent decades with a rapidly growing number of international branch campuses appearing on the scene. This study investigates the economic, cultural and institutional, and educational determinants of transnational higher education on both the extensive margin (number of international branch campuses), and the intensive margin (the total number of educational programmes offered). Using the gravity equation, we applied fixed-effect empirical methods to a panel dataset that combined and extended the raw data from campuses and master’s programmes in 33 source countries and 76 host countries in the period from 1948 to 2016. Estimates reveal that although cultural, economic and institutional ties foster cross-border educational relationships, their effect differs significantly from one margin to another. The study highlights the relevance of globalisation, research activities, and aggregate demand in international higher education.

## Introduction

On 12th May 1551, the Spanish King Charles V signed a royal decree which chartered the first University of the Americas: the National University of San Marcos in Lima, Peru. This constituted one of the earliest examples of transnational education, understanding the term *transnational higher education* (THE) to mean “the mobility of an education program or higher education institution/provider between countries” as used by Knight (2016, p. 36). Since then, the international evolution of higher education (HE) has been closely related to globalisation and the international expansion of economic activity through trade, and to cultural, ethnological and political relations between countries. In recent decades, universities have crossed borders with international branch campuses (IBCs) which operate as an extension of a university, offering HE services in a foreign country.

**Fig. 1:**
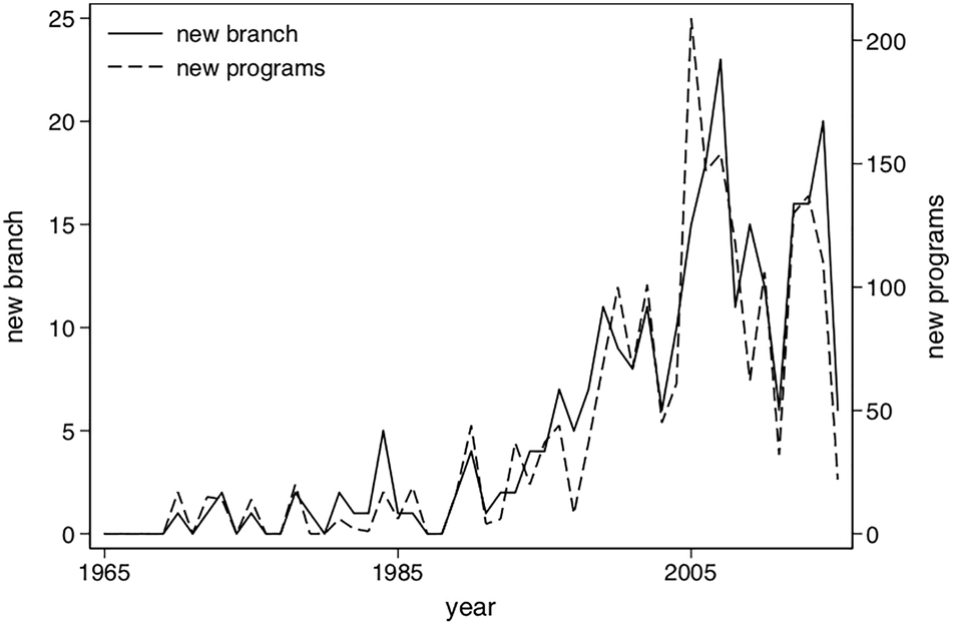
IBC margins: New branches and educational programs openings, per year, 1965–2015. Source: Own calculation with C-BERT data. *Note* The number of IBC prior to 1979 is zero

The expansion of universities is positively associated with higher regional economic growth (Valero and Van Reenen, 2019). It is therefore of social and economic interest to understand how universities expand internationally. On one hand, universities can expand internationally by opening new subsidiaries (extensive margin). On the other, they can grow by offering more programmes in their existing IBCs (intensive margin). As Fig. 1, shows, both margins follow a similar trend, but they are not perfectly correlated, opening up room for research.

Growth in the number of IBCs has been one of the most striking developments in the internationalisation of HE since the turn of the century (Healey, 2015). As of 2017, HE institutions in 33 countries had chartered 249 IBCs in 76 host countries. IBCs have increased by 26% solely in the last 5 years. The trend of IBCs and educational programme openings from 1965-2015 is shown in Fig. 1.

HE growth over the last six decades coincides with the IBCs development, mainly due to massive worldwide educational expansion, driven by worldwide nation-building efforts during the second half of the twentieth century, particularly in former colonies and emerging countries looking to ensure access to tertiary educational opportunities, and most recently, due to a global response to the knowledge economy as well as technological changes which have caused massive higher education internationalisation (Zapp, 2017).

The main academic contribution of the paper is the estimation of the economic, cultural, institutional and educational determinants of the international expansion of universities under a methodologically sound umbrella. The paper uses the workhorse of empirical studies on international economic flows: the gravity equation. This model has been applied to many of the dyadic flows between country pairs (e.g., trade, foreign direct investment, migration, tourism). The study also offers some interesting insights that might be useful for policymakers in their efforts to promote IBCs. Additionally, university management could take advantage of some of the insights in the paper in their international endeavours.

The rest of the paper is structured as follows: “Background and determinants” offers a succinct background on the determinants of IBCs; “Method: The gravity equation” describes the empirical method; “Results and discussion” reports and discusses the results; and, “Conclusions” offers some conclusions.

## Background and Determinants

### International Branch Campuses

IBCs represent one the most tangible examples of THE and have become an increasingly popular form of HE internationalisation, drawing the attention of scholars (Healey, 2014; Wilkins and Huisman, 2012, 2021; Wilkins, 2017; Wilkins and Huisman, 2015). In 2019, Escriva-Beltran et al. reviewed the IBC literature and showed how it spans nine broad areas: (1) Institutional reasons to establish an IBC, (2) IBC models, (3) Student issues, (4) Academic staff issues, (5) Managerial issues, (6) Educational hubs, (7) Sustainability, (8) Language: English as the *lingua franca* and (9) International business.

The closest branch of IBC literature to our analysis is international business literature. To establish links between IBCs and international business, scholars have largely turned to the foreign subsidiaries of multinational corporations (Bhanji, 2008; Healey, 2015; Girdzijauskaite and Radzeviciene, 2014; Lane and Kinser, 2011; Shams and Huisman, 2012). However, Escriva-Beltran et al. (2019) show that the literature examining the determinants and implications of internationalisation flows in HE through the establishment of IBCs is still scarce. They specifically recommend new research into “the economic determinants of IBCs in both countries [and] foreign direct investment” (p. 513).

However, HE scholars highlight the relevance of introducing countrywide cultural and educational dimensions (Volet and Ang, 2012). These dimensions are important to understand how regions meet the needs of the global competitive economy through educational variables such as education systems, university education and knowledge transfer (Cheung and Chan, 2010).

### IBC as Foreign Direct Investment

There is little academic consensus as to whether IBCs should be framed as trade in services or FDI. The gravity equation has been applied extensively to trade in services and FDI flows (Kimura and Lee, 2006; Kleinert and Toubal, 2010). The empirical toolkit is similar in both cases, with the difference lying in the conceptual road to gravity.

On the one hand, the OECD’s benchmark definition of FDI, which is accepted and compatible with International Monetary Fund (IMF) and World Bank statistics, identifies FDI as ‘a category of investment that reflects the objective of establishing a lasting interest by a resident enterprise in one economy (direct investor) in an enterprise (direct investment enterprise) that is resident in an economy other than that of the direct investor’ (OECD, 2008, p. 234).

On the other hand, the IMF considers education services as: ‘services relating to education, such as correspondence courses and education via television or the Internet, as well as by teachers and so forth who supply services directly in host economies.’ IMF (2009, paragraph 10.169).

Many IBCs are in a grey area with relevant quantitative similarities to trade in services. Less investment is required to establish an IBC than other types of investment projects, especially when spaces are rented, non-tenure track professors are hired, and local lecturers are contracted to teach IBC courses. However, the benchmark definition quantifies the FDI in relative terms: a numerical threshold of ownership of 10 percent or more of voting stock.

The focus of this paper is to understand the determinants of IBC flows (the establishment of new IBCs), rather than the specific services offered by these IBCs. Therefore, since universities have a lasting interest and control over IBCs operating in a host country, both in financial and educational terms, our study places IBCs in the frame of FDI.

In this line of work, IBCs are compared to a business that invests in new markets. Therefore, IBCs are studied under similar conceptual grounds as foreign direct investment by international businesses (Guimon, 2016; Ross, 2008). These studies focus on organisation-specific capabilities (managerial and financial capacity), reputation, brand protection and organisational culture as well as government policies.

The empirical applicability of the gravity equation to IBCs is grounded in the research of scholars who have studied FDI and knowledge flows. Bergstrand and Egger (2007) developed a knowledge-and-physical-capital model for international trade flows, FDI and knowledge. Kleinert and Toubal (2010) derived a gravity equation for foreign affiliate sales, and Keller and Yeaple (2013) showed that gravity forces are behind the flow of ideas (patents) across borders. Cuadros et al. (2016) developed and estimated a model for greenfield investment including information asymmetries and migration flows. IBC flows can be modelled in similar terms, since they involve a flow of both foreign capital and knowledge.

### Economic Determinants

Several studies have highlighted the relevance of including economic variables in the analysis of THE (Ahmad and Buchanan, 2017; Abbott and Ali, 2009). The baseline economic specification of the gravity equation for international trade and investment includes GDP and distance as variables that measure demand and transaction costs, respectively. These variables have been used in tangent HE studies. Levatino (2015) showed that countries with a larger population and a higher GDP send more students abroad, whereas distance discourages student mobility. Dobos (2011) documented the inherent problems related to the quality and success of programmes provided by distant offshore partners.

We considered two additional economic controls related to trade and investment readiness between pairs of countries: Bilateral Investment Treaties (BITs) and Regional Trade Agreements (RTAs). BITs are agreements between two countries regarding the promotion and protection of investments made by investors from the paired countries in each other’s territories. RTAs are defined as any agreement involving tariffs lower than most-favoured-nation rates (DiCaprio et al., 2017). In particular, BITs and RTAs are often used as a relevant measure of bilateral stability and economic integration between pairs of countries. Countries with BITs and RTAs in force provide stable mechanisms to arbitrate investment and commercial disputes, which foster international investment and trade (Myburgh and Paniagua, 2016).

### Cultural and Institutional Determinants

Managing an IBC is a complex activity that requires an effort to adapt to cultural, legal and environmental conditions which differ considerably from those on the home campus (Harding and Lammey, 2011; Shams and Huisman, 2012). These include, for example, understanding and balancing the conflicting needs of multiple stakeholders in order to effectively accommodate local needs while operating across multiple cultures (Altbach, 2002b; Healey, 2018; Owens and Lane, 2014; Tierney and Lanford, 2015).

Therefore, IBCs have to face not only the problems posed by geographical distance but also by cultural and institutional distance. Wilkins and Huisman (2012) used institutional theory to explain how regulatory, legal and cultural structures and processes influence the transnational strategies of HE institutions. They stressed that both cultural distance and institutional distance play an important role in transnational activities. Following this strand of literature, Phillips et al. (2009) extended the concept of institutional distance to include institutional uncertainty and thus suggestions for different strategies to establish IBCs.

Eldridge and Cranston (2009) used Hofstede’s national cultural value dimensions as an analytical tool and suggested that national culture affects both the academic and operational management of THE programmes. Their empirical study found that national cultural differences create complications in terms of teaching, assessment procedures and the social aspect of THE programmes. Therefore, THE institutions might seek hosts with cultural, religious and language similarities (Ahmad and Buchanan, 2016).

A common language also reduces cultural and institutional distances. The importance of language in this type of developments is another issue which has been studied by different scholars (Altbach, 2002b; Healey, 2015; Owens and Lane, 2014; Tierney and Lanford, 2015). The economic, political and cultural dimensions of globalisation have promoted the use of English as a language for communication among individuals or groups who do not share a common tongue. English as the *lingua franca* in science and international HE undoubtedly benefits countries such as the United States, UK and Australia, yet increasingly, institutions in non-English speaking countries, such as the Netherlands, are establishing branches overseas with programmes delivered in English (Wilkins and Urbanovic, 2014). Nowadays, students in host countries believe that English language skills are essential in the labour market, especially among multinational employers (Lee, 2014).

Accordingly, we can state that the cultural and institutional determinants of IBCs include a common language, a common administrative border, colonial links and a common religion. These variables have been extensively used in FDI gravity models (Myburgh and Paniagua, 2016; Paniagua and Sapena, 2014).

### Educational Determinants

One relevant set of IBC determinants are specific transnational educational variables such as enrolment, educational expenditure and research activity. Wei (2013) highlighted the importance of a global approach towards educational determinants in THE. Other scholars explained the upswing of IBCs in many developing countries due to strong governmental involvement in supporting and financing these ventures (Ahmad and Buchanan, 2016; Alam et al., 2013; Knight, 2013; Chan and Ng, 2008). Seeber et al. (2016) revealed that educational organisations oriented towards research activity are more likely to conceive the strongest benefits of internationalisation for research.

## Method: The Gravity Equation

### The Gravity Equation for IBCs

Gravity fits international economic flow data well and facilitates the inclusion of real-world features such as multiple countries and trade costs. Therefore, the gravity equation is widely used in empirical research and successfully explains a variety of dyadic economic interactions, such as trade, FDI, migration, tourism, and foreign employment.^1^ Several researchers in HE have used the gravity equation to model international student flows. For example, González et al. (2011) applied the gravity equation to estimate the determinants of student mobility in the Erasmus programme, Bessey (2012) studied international student migration in Germany.

The first attempt to study IBC determinants using the gravity equation appeared in Lien and Keithley (2020). This is our closest reference in literature, though we depart from it and improve the authors’ analysis in four major ways. Firstly, we framed our conceptual analysis within foreign direct investment (FDI) rather than in trade in services. Establishing an IBC is often a greenfield investment in a foreign country involving tacit knowledge transfer, rather than the export of coded knowledge. Therefore, IBCs represent the persistent, long-lasting footprint of a foreign university in a foreign country.

Secondly, we investigated the determinants of both intensive and extensive margins, as is customary in gravity literature, based on the work by Chaney (2008).^2^ Organisations can grow internationally by opening new IBCs or by offering more programmes abroad. This distinction is particularly interesting since the interpretation of the determinants of both margins reveals different insights. The extensive margin captures the factors behind the creation of new IBC partners (i.e., how many); whereas the intensive margin reveals the factors behind the volume of IBCs (i.e., how large).

Thirdly, we addressed several empirical caveats by addressing sample selection bias, omitted variable bias, and unobservable heterogeneity, which we will discuss in the empirical section in detail. Lastly, we extended the sample period to 69 years of data.

### Estimating Gravity

Estimating gravity equations presents several known caveats (Baldwin and Taglioni, 2006; Yotov et al., 2016). These include zeros in the dependent variable, hetokedasticity in the error term, multilateral resistance terms, unobserved heterogeneity, omitted variable bias, and sample selection bias. We followed an empirical methodology aimed at correcting these well-known issues.^3^

Dynamic dyadic data (e.g, time-varying country-pair trade, FDI and migration data) are typically characterised by numerous zeros, which indicate that a pair of countries did not invest or trade in a particular year. IBC data are no different and contain many zeros, as seen in the descriptive statistics presented in Table 1. Therefore, in line with other empirical studies, we used a similar non-linear specification of the gravity equation (Cuadros et al., 2016; Myburgh and Paniagua, 2016; Paniagua and Sapena, 2014). In particular, we estimated a non-linear variant of the gravity equation with the Poisson Pseudo-maximum Likelihood (PPML) estimator, which is similar to the one proposed by Silva and Tenreyro (2006). PPML corrects heterokedasticity in the error term and offers consistent estimates of data with zeros since this estimator does not require log linearisation of the variables. In particular, the equation to estimate for each margin is^4^:

1 $$\begin{aligned} \left\{ IBC_{ijt},master_{ijt}\right\}= & {} \exp \left( \begin{array}{c} Economic_{ijt}\\ Cultural_{ijt}\\ Educational_{ijt}\\ +\lambda _{i}+\lambda _{j}+\lambda _{t} \end{array} \right) +e_{ijt}, \end{aligned}$$

where the dependent variable is either the number of IBCs ($IBC_{ijt}$) or the master’s programmes $master_{ijt}$ in the home country *i* in host country *j* in year *t* (i.e., IBC flows) and $e_{ijt}$ represents a stochastic error term. The dependent variables were divided into three different sets of economic, cultural and educational determinants, following our previous discussion. The variable definition, sources, summary statistics and year coverage are reported in Table 1.^5^ Non-dyadic variables were introduced independently for host and source countries to distinguish between pull and push factors.

**Table 1:** Variable description, sources and descriptive statistics

Variable	Mean	s.d.	max	min	Description	Source	Years
IBC	0.001	0.052	13	0	Number of international branch campus	C-BERT	1979–2016
Masters	0.008	0.506	151	0	Number of masters programs offered in IBC		
Economic determinants							
GDP	22.653	2.501	30.555	15.993	Gross domestic product (log)	World Bank	1948–2016
Distance	8.814	0.813	9.897	0.004	Distance in kilometres between country capitals	CEPII	
BIT	0.032	0.177	0	1	Takes the value 1 when countries have a Bilateral Investment Treaty in force		
RTA	0.030	0.172	0	1	Takes the value 1 when countries have a Regional Trade Agreement in force		
Cultural and institutional determinants							
Border	0.012	0.110	1	0	Takes the value 1 when countries share a common border, and 0 otherwise	CEPII	1948–2016
Language	0.174	0.379	1	0	Takes the value 1 if both countries share the same official language		
Colony	0.010	0.099	1	0	Takes the value 1 if the two countries have ever had a colonial link, and 0 otherwise		
Religion	0.174	0.249	1	0	Takes the value 1 if the two countries have the same majority religion, and 0 otherwise		
Educational determinants							
Enrolment	18.071	14.35	46.263	5.343	School enrolment, tertiary (% gross)	World Bank	1970–2016
Expenditure	8.347	2.137	13.703	2.691	Government expenditure on education (log)		1999–2016
Articles	5.51	3.12	12.99	-2.30	Scientific and technical journal articles (log)		2003–2016

Notes: The first IBC dates from year 1979. The variable IBC is zero from 1948 to 1979

We used a set of country fixed dummies as control variables to monitor for any unobserved characteristics at country level ($\lambda _{i}+\lambda _{j}$). These variables are standard in gravity models and absorb the effect of any country characteristics. In the specialized literature, they are known as multilateral resistance terms and their omission biases gravity estimates (Anderson and Van Wincoop, 2003). Conceptually, these terms capture remoteness, or the fact that IBCs could be located in third countries with similar characteristics. These terms absorb all non-dynamic unobserved country characteristics and control the incidence of each country in the world’s IBC transaction costs. In addition, we added a dummy for every year in the sample $\lambda _{t}$ to account for a common trend in world GDP which is not specific to country pairs. These time dummies absorbed the effect of structural changes during the sample period (e.g., globalisation, common technological advances, financial crises).

In total, we introduced 518 dummy control variables: 225 host country dummies, 225 home country dummies and 68 year dummies (whose coefficients have not been included for brevity). These variables appear in canonical gravity models of trade and FDI to avoid estimation bias.

To prevent the bias that stems from constant unobserved heterogeneity and omitted variables at country-pair level, we also estimated a country-pair fixed effects panel. These fixed effects were dummies that controlled for unobservable heterogeneity between country pairs. These variables absorbed the effect of all the variables that were constant between country pairs (distance, common language, border, colony and religion). This specification offers greater precision in the estimates of the rest of variables with a time dimension (BIT, RTA and educational variables) within country pairs. Additionally, Baier and Bergstrand (2007) showed that country-pair fixed effects reduced endogeneity bias.

### Data Sources

Gravity data came from the *Centre d’Etudes Prospectives et d’Informations Internationales* (CEPII). The CEPII provides a gravity dataset for all world country pairs for the period from 1948 to 2016. This dataset was originally generated by Head et al. (2010). The education determinants came from the World Bank and their time span and country scope were not as broad as the rest of the variables. The “Articles” variable referred to the number of scientific and technical journal articles published from the set of journals covered by the Science Citation Index (SCI) and Social Sciences Citation Index (SSCI). Articles were classified by year of publication and assigned to region/country/economy on the basis of institutional address(es) listed in the article.^6^

IBC data was collected from the Cross-Border Education Research Team (C-BERT) dataset. The C-BERT database has a total of 247 IBCs in operation and reported a total of 33 source countries and 76 host countries during the period 1979–2016.

The C-BERT database records two type of data that we used in our analysis: the number of IBCs, which we identified as the extensive margin, and the number of programmes, which we identified as the intensive margin. Our empirical strategy mentioned earlier focuses on both types of measures. However, the data on the extensive margin might more reliable due to the strategic behaviour of universities, in exaggerating successes and hide failures. For example, they claim they are starting a programme even though there are very few students.

The publicly available C-BERT dataset does not record IBC closures. This makes empirical analysis easier since there are no negative values in the dependant variable. Therefore, the results are bound to the static establishment of new facilities. This limitation is not uncommon in FDI studies, especially in those dealing with greenfield or new FDI (Paniagua et al., 2015).

The lack of IBC closure data in this analysis is also relevant for the measurement of left-hand-side variables (IBC number and MA programs). Without the information on IBC closures, measuring IBCs in stocks would bias the estimates towards larger countries that welcomed numerous IBCs, which were carried into the future, even if these IBCs closed afterwards. Additionally, the economics models that derive the gravity equation used flows as a dependent variable due to the market clearing condition that states that all goods are sold and consumed at the end of each period the economics models that derive the gravity equation use flows as a dependent variable due to the market clearing conditions that imposes that all goods are sold and consumed at the end of each period (Anderson, 2011). Stocks ‘carry’ information from previous years that clashes with the market clearing condition.

Our empirical strategy, which was compatible with zeros in the dependent variable, enabled us to extend the analysis to many other countries by adding zeros to instances where there were no IBCs. Thus, we were able to expand the scope of the dataset and minimise the bias stemming from ignoring zeros in gravity models (Paniagua, 2016).

Thus, we could also control for sample selection bias that stemmed from estimating only a treated sample, i.e., only the observations with an IBC. By extending the IBC variable back to 1948, we were able to estimate the effects of the determinants in the sample that included both treatment and control observations.

To prevent zero-inflation issues, we used Stata’s command ppmlhdfe by Correia et al. (2020). This command uses a fast and efficient PPML estimation method that deals with the inclusion of many high-dimensional fixed effects and zeros. The correlation matrix is shown in Table 2. We did not observe a high pair-wise correlation among the variables that could have induced multicollinearity bias in the analysis.

**Table 2:** Correlation matrix

	IBC	Masters	GDP	Distance	BIT	RTA	Border	Language	Colony	Religion	Enrolment	Expenditure
IBC	1											
Masters	0.708$$^{***}$$	1										
GDP	0.0597$$^{***}$$	0.0398$$^{***}$$	1									
Distance	− 0.00732$$^{***}$$	− 0.00699$$^{***}$$	− 0.0503$$^{***}$$	1								
BIT	0.0321$$^{***}$$	0.0233$$^{***}$$	0.238$$^{***}$$	− 0.122$$^{***}$$	1							
RTA	0.0317$$^{***}$$	0.0213$$^{***}$$	0.156$$^{***}$$	− 0.215$$^{***}$$	0.210$$^{***}$$	1						
Border	0.0233$$^{***}$$	0.0107$$^{***}$$	0.0354$$^{***}$$	− 0.286$$^{***}$$	0.0511$$^{***}$$	0.126$$^{***}$$	1					
Language	0.00598$$^{***}$$	− 0.000227	− 0.103$$^{***}$$	− 0.0582$$^{***}$$	− 0.0125$$^{***}$$	0.0367$$^{***}$$	0.0795$$^{***}$$	1				
Colony	0.0423$$^{***}$$	0.0301$$^{***}$$	0.0894$$^{***}$$	− 0.0503$$^{***}$$	0.0883$$^{***}$$	0.0472$$^{***}$$	0.0902$$^{***}$$	0.118$$^{***}$$	1			
Religion	− 0.00445$$^{***}$$	− 0.00304$$^{***}$$	− 0.0127$$^{***}$$	− 0.191$$^{***}$$	0.0115$$^{***}$$	0.0741$$^{***}$$	0.0942$$^{***}$$	0.198$$^{***}$$	0.0446$$^{***}$$	1		
Enrolment	0.0269$$^{***}$$	0.0247$$^{***}$$	0.140$$^{***}$$	− 0.0291$$^{***}$$	0.227$$^{***}$$	0.226$$^{***}$$	− 0.00340$$^{***}$$	− 0.102$$^{***}$$	0.0603$$^{***}$$	− 0.0136$$^{***}$$	1	
Expenditure	0.0340$$^{***}$$	0.0297$$^{***}$$	0.0231$$^{***}$$	− 0.0236$$^{***}$$	0.235$$^{***}$$	0.136$$^{***}$$	0.0324$$^{***}$$	− 0.102$$^{***}$$	0.107$$^{***}$$	0.0263$$^{***}$$	0.573$$^{***}$$	1
Articles	0.0405$$^{***}$$	0.0293$$^{***}$$	0.0134$$^{***}$$	− 0.0743$$^{***}$$	0.275$$^{***}$$	0.169$$^{***}$$	0.0406$$^{***}$$	− 0.109$$^{***}$$	0.0902$$^{***}$$	− 0.0399$$^{***}$$	0.655$$^{***}$$	0.928$$^{***}$$

*$$p<0.05$$, $$^{**}$$$$p<0.01$$, $$^{***}$$$$p<0.001$$

## Results and Discussion

### Branch Campuses (Extensive Margin)

The results reported in Table 3 show the PPML estimation of the determinants of the extensive margin. It is worth starting with the interpretation of our baseline estimates in column 1. As expected, the gravity equation fitted the data well with a pseudo- $R^{2}$ of 0.7. Overall, the variables had the expected signs, and most were significant to a level of 0.01 (except language $p<0.1$ and religion $p<0.05$).

**Table 3:** Results:Extensive margin (number of IBCs)

	(1)	(2)	(3)	(4)	(5)
log GDP home	0.043	− 0.012	− 0.050	− 0.014	− 0.329$$^{**}$$
	(0.08)	(0.06)	(0.13)	(0.16)	(0.17)
log GDP host	0.691$$^{***}$$	0.689$$^{***}$$	0.266$$^{**}$$	0.193	− 0.083
	(0.06)	(0.04)	(0.10)	(0.12)	(0.12)
BIT dummy	0.253$$^{***}$$	0.554$$^{***}$$	− 0.060	− 0.280	0.494$$^{**}$$
	(0.05)	(0.10)	(0.21)	(0.25)	(0.21)
RTA dummy	0.696$$^{***}$$	0.369$$^{***}$$	0.308$$^{**}$$	− 0.150	0.249$$^{**}$$
	(0.06)	(0.10)	(0.14)	(0.11)	(0.11)
log Education Expenditure home			0.521$$^{*}$$	0.987$$^{***}$$	1.081$$^{**}$$
			(0.29)	(0.37)	(0.44)
log Education Expenditure host			− 0.103	0.092	0.255$^{*}$
			(0.12)	(0.13)	(0.14)
Tertiatry School enrollment (%) home				− 0.022$$^{***}$$	− 0.028$$^{***}$$
				(0.01)	(0.01)
Tertiatry School enrollment (%) host				0.004	0.010$$^{**}$$
				(0.00)	(0.00)
log Articles home					0.768$$^{***}$$
					(0.22)
log Articles host					0.138$$^{**}$$
					(5.13)
log Distance	− 0.547$$^{***}$$				
	(0.03)				
Contiguity	0.493$$^{***}$$				
	(0.08)				
Common language	0.149$^{*}$				
	(0.09)				
Common majority religion	0.386$$^{**}$$				
	(0.17)				
Colonial relationship	1.602$$^{***}$$				
	(0.08)				
Observations	1,251,210	1,267,368	188,907	119,663	84,533
Pseudo-$R^{2}$	0.703	0.920	0.930	0.345	0.346
Country FE	Yes	No	No	No	No
Country-pair FE	No	Yes	Yes	Yes	Yes
Year FE	Yes	Yes	Yes	Yes	Yes

Robust standard errors in parentheses, clustered by country pair

$$^{*}$$$$\hbox {p}< 0.1$$, $$^{**}$$$$p< 0.05$$, $$^{***}$$$$p< 0.01$$

#### Economic Determinants

In the economic determinants, host GDP was a strong pull factor as expected. A 1% increase in the host’s GDP grew the number of IBCs by 0.7% on average. However, the GDP of the home country was not significant at any standard level. This means that the economic pull of demand was a host phenomenon. The economic activity of the home country had no significant effect on determining the extensive margin of IBCs, meaning that the creation of cross-border educational links was solely determined by host demand. The HE literature generally supports the idea that local demand for IBCs exists in order to foster and drive economic development, growth and wealth in the host countries (Altbach, 2002a; Kosmützky, 2018; Miller-Idriss and Hanauer, 2011; Wilkins and Huisman 2012).

Distance, which captures transaction costs, such as information and trade costs, had a negative and significant effect. All other things considered, countries which were farther away had fewer IBCs. This result is in line with the vast majority of gravity estimates of dyadic data (Disdier and Head, 2008). An expatriate faculty member at an IBC is one of the factors of IBC success. Moving faculty members abroad is expensive and increases IBC tuition fees, but reinforces the idea of students receiving the same education as at the home university and helps to build a global university brand, underscoring international, premium quality education (Healey, 2018; Wilkins and Neri, 2019).

Economic integration via bilateral agreements also proved to be a relevant determinant of branch campuses. The RTA coefficient was 0.696, which means that pairs of countries that had signed RTAs had, on average, 93% more IBCs than those which had not.^7^ Investment treaties had a more moderate coefficient (0.253), meaning that the impact brought an average increase of 23%. However, the effect of BITs and RTAs on IBCs was higher than their effect on Greenfield FDI flows. Myburgh and Paniagua (2016) reported a coefficient of 0.050 for BITs and 0.183 for RTAs on the extensive margin of FDI flows in a large sample of countries.

RTA and BITs aim to encourage trade and FDI flows from high-income countries to lower-income hosts by assuring legal standards for investors. There is a wide range of estimated effects on FDI in the literature; Bellak (2015) reported a wide range of BIT estimates on FDI, including positive, non-significant and negative (11% in total) estimated coefficients. Falvey and Foster-McGregor (2017) summarised how the literature responded to this ambiguity, concluding that BITs appear to have no impact upon FDI flows for country-pairs that are too dissimilar in terms of the strength of their political institutions. Therefore, the larger resemblance of home and host countries in IBC investment might explain their higher impact on IBC than aggregate FDI flows.

However, when we introduced country-pair fixed effects in column 2, the effect of BITs increased to 74% and the effect of RTAs dropped to 44%. IBCs are a lasting investment relationship and therefore the estimates should have resembled the expected effect of these variables on FDI rather than on trade. This result highlights the importance of introducing country-pair fixed effects in the gravity equation to estimate the effect of time-varying variables (Baier and Bergstrand, 2007). That is why when we turn to the education determinants in columns 3–5 we directly reported the results with country-pair fixed effects.

Overall, the economic determinants were aligned with previous findings in the literature discussed earlier. However, we did find some differences compared to the work of Lien and Keithley (2020). For example, they reported a non-significant effect of common language. In addition, we found that host GDP had no effect on IBCs whilst these two authors reported a positive result on host GDP (though it was lower than home GDP). In trade models, both GDPs should have a similar and positive effect. However, Kleinert and Toubal (2010) used a factor-proportions theory to show that this is not always the case for FDI, where the expected sign of the source GDP can be negative. These results highlight the relevance of interpreting IBCs as FDI as opposed to trade in services and of adequately controlling for all aforementioned biases.

We performed further tests (not reported here for succinctness) and found that the year dummies were responsible in most part for absorbing the home GDP effect. This suggests that the common trend in the growth of the world’s GDP (e.g., globalisation controlled by time dummies) explained the effect of home economic growth on IBCs. This does not imply that the economic growth of the country was not positive for IBCs, but rather that it was not something specific to the home country but rather a general trend across all country pairs.

#### Cultural and Institutional Determinants

Our analysis confirmed the hypothesis that the cultural and institutional context was relevant. Countries that shared a common language had 16% more IBCs on average, in line with the meta-analysis of the effects of a common language in trade (Egger and Lassmann, 2012) and in HE literature (Altbach and Knight, 2007; Wilkins and Urbanovic, 2014).

Having the same majority religion had a similar effect on the number of IBCs: countries had 47% more IBCs from countries with the same majority religion. However, the largest effect was colonial links, as Head et al. (2010) had previously highlighted for trade. Countries which shared a historical colonial relationship had on average five times more IBCs than those that did not, in line with earlier results in the literature. Moreover, Li and van Baalen (2007), Shams and Huisman (2012) or more recently Healey (2018), defended that the greater the distance in the cultural, societal, and regulatory distance between countries, the stronger the pressure to maintain academic integration. Cultural distance imposes a challenge in the management equilibrium between home universities seeking to build their global brand and local IBCs.

The positive estimate of the contiguity variable suggests that countries that shared a common administrative border (e.g., Canada and USA), had on average 63% more IBCs than similar country pairs without a border.

#### Educational Determinants

In terms of educational determinants, columns 3–5 of Table 3 show a wide range of educational push and pull factors. Most of the variables were significant in the home country, revealing that most educational determinants acted as push factors. On the one hand, the most relevant economic determinant (GDP) was exclusively a pull factor (i.e., it was only significant in the host country). On the other hand, educational determinants (with the exception of scientific articles) were push factors (i.e., they were only significant in the home country). This result sheds some light on the difference between economic and educational determinants. What is relevant from an economic perspective is high demand (e.g., students willing to pay tuition fees). However, from an educational perspective, the importance lies in the home country’s ability to create organisations that have the educational muscle to cross borders.

This conclusion can be better understood by observing the educational determinants in detail. The log of tertiary educational expenditure was positive and significant in all cases, with the marginal effects ranging from 0.521 ($\hbox {p}< 0.1$) to 1.081 ($\hbox {p}< 0.05$). The results in the last two columns suggest that the elasticity of expenditure was close to one, meaning that an increase (or decrease) in the home country’s expenditure on tertiary education led to an equivalent change in IBCs (in percentage terms). Conversely, host expenditure was not significant in columns 3 and 4, and only appeared as positive and mildly significant after controlling for school enrolment and scientific articles in column 5. This result has clear policy implications when countries wish to promote IBCs and foreign programmes in their territory, in line with the conclusions of Seeber et al. (2016).

The statistical significance of tertiary school enrolment revealed a similar pattern. It was only significant (and positive) as a pull factor after controlling for the rest of the educational variables and it was robustly significant (and negative) throughout columns 4 and 5 as a push factor. In this case, the elasticity of the home country’s tertiary education was negative, which can be interpreted as a response to demand conditions at home. The fewer (more) the students universities find at home, the greater (lesser) their international activity. Therefore, after controlling for other factors, the evidence empirical suggests that IBCs acted as fishing nets for international students when demand at home was weak. The positive sign of the host’s tertiary education seems to be aligned with this interpretation, since an increase in the number of foreign students fostered IBCs.

Lastly, scientific production acted simultaneously as a push and pull factor. The countries that increased their scientific production on average grew their outbound and inbound IBCs, supporting the arguments of scholars who suggest that internationalisation has a positive influence on universities’ reputation, research quality, teaching quality and graduate employability. IBC host countries are expected to increase local demand for HE, improve the quality of HE, and undergo rapid development in the volume of publications they put out. Success in an academic career depends on publishing in international peer-reviewed journals. The challenge for an IBC is to attract academic faculty aligned to the research agendas of the home university and with an interest in focusing their research on issues of local impact and interest (Altbach and Knight, 2007; Delgado-Márquez et al., 2013; Healey, 2018; Pohl and Lane, 2018).

### Masters Programs (Intensive Margin)

Once we had studied the determinants of the number of branch campuses, we analysed what determined the volume or size of these IBCs. The same effort may not be required to open a foreign campus with just one programme as working with many programmes. We chose to focus on master’s programmes as a good indicator of the volume, capital invested and number of students in IBCs.

#### Economic Determinants

The estimates of the determinants of the number of educational programmes (intensive margin) are reported in Table 4. Again, most of the coefficients showed the expected signs and statistical significance and the pseudo-$R^{2}$ was high. Distance, contiguity, RTAs, BITs and colonial relationship had the same magnitudes (in statistical terms) as the extensive margin estimates. Therefore, we focused on interpreting the variables which were different.

**Table 4:** Results: Intensive margin (Masters programs of IBCs)

	(1)	(2)	(3)	(4)	(5)
log GDP home	− 0.414$$^{***}$$	− 0.366$$^{***}$$	− 0.598$$^{***}$$	− 0.496$$^{**}$$	− 0.477$$^{*}$$
	(0.13)	(0.09)	(0.20)	(0.22)	(0.27)
log GDP host	0.704$$^{***}$$	0.792$$^{***}$$	0.214	0.203	0.429$$^{*}$$
	(0.10)	(0.08)	(0.18)	(0.20)	(0.23)
BIT dummy	0.473$$^{***}$$	0.085	0.874$$^{***}$$	1.416$$^{***}$$	0.484$$^{**}$$
	(0.08)	(0.12)	(0.24)	(0.32)	(0.20)
RTA dummy	0.721$$^{***}$$	0.037	0.021	0.265$$^{**}$$	0.501$$^{***}$$
	(0.08)	(0.09)	(0.15)	(0.12)	(0.16)
log Education Expenditure home			0.856$$^{**}$$	0.842$$^{*}$$	0.864
			(0.41)	(0.50)	(0.58)
log Education Expenditure host			− 0.139	$-$0.003	− 0.313
			(0.20)	(0.23)	(0.26)
Tertiatry School enrollment (%) home				− 0.039$$^{***}$$	− 0.041$$^{***}$$
				(0.01)	(0.01)
Tertiatry School enrollment (%) host				− 0.007	− 0.006
				(0.00)	(0.00)
log Articles home					0.436$^{*}$
					(0.26)
log Articles host					0.614$$^{***}$$
					(0.19)
log Distance	− 0.554$$^{***}$$				
	(0.05)				
Contiguity	0.878$$^{***}$$				
	(0.12)				
Common language	− 1.246$$^{***}$$				
	(0.13)				
Common religion	1.212$$^{***}$$				
	(0.21)				
Colonial relationship	1.163$$^{***}$$				
	(0.11)				
Observations	1,251,210	1,267,368	188,907	119,663	84,533
Pseudo-$R^{2}$	0.827	0.903	0.924	0.346	0.332
Country FE	Yes	No	No	No	No
Country-pair FE	No	Yes	Yes	Yes	Yes
Year FE	Yes	Yes	Yes	Yes	Yes

Robust standard errors in parentheses, clustered by country pair

$$^{*}$$$$\hbox {p}< 0.1$$, $$^{**}$$$$\hbox {p}< 0.05$$, $$^{***}$$$$\hbox {p}< 0.01$$

The first difference in the number of IBCs was that GDP acted as both a push and a pull factor. Let us recall that when we studied the full set of educational determinants of IBCs, home GDP was negative (last column in Table 3). This result is in line with Kleinert and Toubal’s (2010) factor-proportions theory, which suggests that FDI increases when economic activity declines in the home country. Intuitively, firms seek economic opportunities abroad as a response to economic decline at home. We interpreted this as a demand-driven model of IBC programmes: increasing host economic activity nearly doubled the the marginal effect of decreasing home GDP.

The higher effect of bilateral treaties (RTAs and BITs) on the number of programmes was in line with the results reported by Myburgh and Paniagua (2016) on the intens ive margin of FDI flows in a large sample of countries. These authors found mixed coefficient results for BITs (0.092 being the higher estimate) and 0.459 for RTAs. A plausible reason for these results is found in Paniagua et al. (2015), who showed that the effectiveness of these treaties was related to the size of investment projects. HE seems to be following the same kind of pattern as that documented by FDI literature.

#### Cultural and Institutional determinants

A common language, which was significant and positive in the extensive margin, had a negative and significant effect on the number of programmes. The rationale was that sharing a language triggered an IBC, but it decreased the number of master’s programmes. These results could be driven by foreign campuses which offered English programmes in country pairs with a different official language (e.g., French or Spanish) (Ahmad and Buchanan, 2016; Altbach and Knight, 2007; Altbach, 2007; Wilkins and Urbanovic, 2014).

As Altbach (2007) indicated, English is the imperial tongue that dominates academia and business and, at this level of education, English matters. Accordingly, European universities have introduced teaching in English over the last few years as a means to achieving internationalisation objectives such as improving the employability of their graduates, and facilitating international exchange activities as Tatzl (2011) and Wilkins and Urbanovic (2014) demonstrated. Continuing with this idea which our results confirmed, Wilkins (2010) put improving English at the same level as enhancing employment prospects, as well as experiencing a different culture, as the main motivations to study overseas.

#### Educational Determinants

Columns 3–5 in Table 4 show that the influence of educational variables on the number of educational programmes resembled that of the number of IBCs. Expenditure on tertiary education at home was a strong push factor, as it was positive and significant (however it was not significant with the full set of controls in column 5). As described above, host expenditure on HE was not a relevant pull factor. Tertiary school enrolment followed the same pattern: it was negative and significant at home and not significant in the host country. Scientific production via published articles acted both as a push and pull factor. However, conversely to the case of IBC numbers, articles in the host country had a higher order of magnitude than at home. This means that research undertaken in host countries was a more relevant factor (in relative terms) to determine the volume of IBCs than their location.

## Conclusions

This study has analysed the economic, institutional and educational determinants of the extensive and intensive margins of IBCs. To this end, we used the gravity equation to study the number of IBCs and programmes across a wide range of countries and time spans. Our empirical specification enabled us to hedge several estimation biases. Our results are interesting in many ways and reveal that IBCs share certain common traits with other economic flows, and more specifically FDI, but also exhibit some very distinctive traits. The estimates highlight the relevance of analysing both margins to obtain a full picture of the determinants of IBCs. Otherwise, we could be led to believe that IBCs expand in countries which share a common language when, in fact, our estimates show that this is the case in the extensive margin where IBCs offer fewer programmes.

The paper shows that it is appropriate to frame IBCs within the FDI theory, which can explain the negative sign of host GDP. This might also be relevant for policy reasons, as most countries have separate investment and export promotion agencies. The services and skill sets required to deliver them are different. For example, investment promotion agencies (IPAs) offer aftercare services for multinational affiliates. Including IBCs within the range and radar of IPAs would open up a new playing field for both universities willing to cross borders and countries seeking international education projects.

Our study has interesting policy implications both for policymakers and university management in source countries. Governments and HE management interested in promoting the international expansion of their universities could look into policies aimed at increasing scientific production. This could be done, for example, by granting publication incentives.

Additionally, these kinds of incentives would increase growth in both the number of IBCs and their programmes abroad. Increasing scientific production would also increase university reputations in terms of rankings that include scientific articles in their indices. Nonetheless, these indices are more important in Science, Technology, Engineering and Mathematics (STEM) disciplines. Therefore, university policies aimed at increasing STEM master’s programmes in IBCs could increase both the number of IBCs and their position in university rankings. All other push factors, except educational expenditure, would not be applicable, as they have undesired effects on the economy or on universities (e.g., decreasing enrolment or income). Regarding host countries, in addition to the standard economic policies fostering economic growth, policymakers have a wider range of educational policy measures to attract and promote IBCs and increase the numbers of their master’s programmes. Policies tailored to increase government expenditure per tertiary student and enrolment might prove useful to foster IBCs. However, to increase the number of master’s programmes the most effective policy measure is to increase scientific production.

Host countries might also find hints in our analysis as to which countries to target. Our results suggest that universities from source countries with a slowing economy increase their master’s programmes in growing host economies. Regional promotion agencies, for example, might use this information to include these universities in their aftercare services.

Some limitations that surface in our study could open a new path for further research. One of the limitations of the gravity approach is that it does not allow us to investigate micro-level determinants. For example, we focused on countrywide determinants rather than on specific university-level determinants. Although both might be highly correlated, research that focuses on the specific university determinants of IBCs is certainly an interesting avenue of research. Our study is among the first to reconcile the gravity model with multilateral resistance terms and THE. Revisiting other aspects of cross-border education, such as international student flows is another area that could be of interest to future scholars. Finally, our results showed that unobservable common trends (globalisation) have an effect on IBCs. The recent COVID-19 pandemic imposes challenges in all areas of our society, especially in those dealing with globalisation and education. How universities will respond to this new situation is still an open, daunting question.

## Footnotes

1 For a survey on gravity models and applications see Anderson (2011).

2 See, for example, Paniagua and Sapena (2014), Myburgh and Paniagua (2016).

3 In the study of IBC determinants, Lien and Keithley (2020) were only able to address two issues: zeros in the dependent variable and heterokedasticity in the error term by using PPML. Additionally, their choice of the dependent variable in stocks, rather than in flows, is hard to justify in a gravity setup since stocks depend on all past flows.

4 Note that in the form by “*Cultural*” authors refer “Cultural and institutional”

5 The reported summary statistics includes zeros. The mean and s.d. for IBC without zeros in 1.52 and 1.29 respectively. The mean and s.d. for masters without zeros were 4.08 and 6.55 respectively.

6 For more information visit the World Bank’s Metadata Glossary: https://databank.bancomundial.org/metadataglossary/environment-social-and-governance-(esg)-data/series/IP.JRN.ARTC.SC

7 Calculated by $$(\exp (\beta )-1)*100$$
